# Dihydrotanshinone Triggers Porimin-Dependent Oncosis by ROS-Mediated Mitochondrial Dysfunction in Non-Small-Cell Lung Cancer

**DOI:** 10.3390/ijms241511953

**Published:** 2023-07-26

**Authors:** Dongjie Zhang, Renyikun Yuan, Jiaping Pan, Qiumei Fan, Kaili Sun, Zhipeng Xu, Xiang Gao, Qinqin Wang, Jia He, Yaqing Ye, Zhengrong Mu, Jing Leng, Hongwei Gao

**Affiliations:** 1 College of Basic Medical, Guangxi University of Chinese Medicine, Nanning 530200, China; 2 College of Pharmacy, Guangxi University of Chinese Medicine, Nanning 530200, China; 3 College of Basic Medical, Guangxi Medical University, Nanning 530200, China

**Keywords:** dihydrotanshinone, Porimin, ROS, mitochondrial dysfunction, oncosis, NSCLC

## Abstract

Lung cancer is one of the leading causes of cancer death. Non-small-cell lung cancer (NSCLC) accounts for the majority of lung cancer diagnoses. Dihydrotanshinone (DHT) is a compound extract from *Salvia miltiorrhiza*, which has favorable anti-inflammatory and anti-cancer activities. However, the role of DHT in NSCLC has not been fully studied. The anti-cancer drugs used for treating lung cancer often lead to apoptosis; however, the drug resistance of apoptosis restricts the effect of these drugs. Oncosis is a passive form of cell death that is different from apoptosis. It is characterized by cell swelling, and Porimin is a specific marker for oncosis. In this study, the role of DHT in mediating oncosis in A549 cells was investigated. In vitro, the MTS assay was used to detect cell activity after DHT treatment. Microscopy and electron microscopy were used to observe cell morphology changes. Western blotting was used to detect protein expression. Flow cytometry was used to detect intracellular reactive oxygen species (ROS) level, calcium ion (Ca^2+^) level, and cell mortality. The intracellular Lactic dehydrogenase (LDH) level was detected by an LDH detection kit after DHT treatment. The ATP level was detected using an ATP detection kit. In vivo, Lewis lung cancer (LLC) xenograft mice were used to evaluate the anti-tumor effect of DHT. Hematoxylin and eosin (HE) staining was used to detect the pathology of lung cancer tumors. The detection of Porimin in the tumor tissues of the mice after DHT administration was assessed by immunohistochemistry (IHC). The results of this study showed that DHT treatment changed the cell morphology; destroyed the mitochondrial structure; increased the expression of Porimin; increased the levels of LDH, ROS, and Ca^2+^; decreased the mitochondrial membrane potential and ATP level; and played an anti-tumor role in vitro by mediating oncosis in A549 cells. The in vivo studies showed that DHT could effectively inhibit tumor growth. The results of protein detection and IHC detection in the tumor tissues showed that the expression of Porimin was increased and that oncosis occurred in the tumor tissues of mice. DHT triggered Porimin-dependent oncosis by ROS-mediated mitochondrial dysfunction in NSCLC. The in vivo studies showed that DHT could inhibit tumor growth in LLC xenograft mice by triggering oncosis. This study indicates the potential for DHT to treat NSCLC.

## 1. Introduction

Lung cancer is one of the most common and most lethal malignancies in the world [1]. It was one of the top ten major cancer types in the United States in 2023 in terms of new cancer cases and deaths estimated by gender. In males, lung cancer accounts for 12% of new cancers and 21% of deaths [1]. In women, lung cancer accounts for 13% of new cancers and 21% of deaths [1]. Lung cancer is relatively rare before the age of 50, after which the risk increases with age [2]. Lung cancer can be divided into small-cell carcinoma (SCLC) and NSCLC. NSCLC is the most common pathological type of lung cancer, accounting for about 80–85% of the diagnoses [1]. At present, the main treatment methods for NSCLC are surgery, chemotherapy, radiotherapy, immunotherapy, and molecular targeted drug therapy [3]. Chemotherapy for NSCLC is targeted and can be tailored to each patient. Platinum-based chemotherapy is one of the most commonly used treatments in clinical practice. Clinically, the combination of platinum and other drugs is often used to treat lung cancer. For example, patients with adenocarcinoma treated with cisplatin/pemetrexed have a higher survival rate than patients treated with cisplatin/gemcitabine [4]. Cisplatin exerts its anti-cancer effects via a variety of mechanisms, the most important of which are DNA damage and apoptosis [5]. However, resistance against cisplatin-induced tumor apoptosis limits the drug’s application in clinical treatment [2]. It has been reported that the treatment of A549 cells with cisplatin results in an increase in cytoplasmic RAP1, and the overexpression of RAP1 can desensitize A549 cells to cisplatin [6]. Therefore, the search for new therapeutic drugs is crucial to improve the treatment and prognosis of NSCLC patients.

Oncosis is a form of non-programmed cell death. The earliest discovery of oncosis was observed by the scientist von Reckling [7], who found that osteocytes underwent swelling death. In 1995, Majno [8] observed cell swelling, blebbing, and karyolysis, accompanied by an inflammatory response, and it is thus named oncosis because of the morphological swelling of cells. Since then, studies on oncosis have flourished. The main causes of oncosis are an increase in cell volume, the swelling of the nucleus and organelles, the destruction of the mitochondrial cristae, increased permeability of the cell membrane, a nuclear solution, an inflammation reaction, etc.

The mechanism of oncosis is not completely clear at present, but previous studies have reported that oncosis is related to ROS and mitochondrial dysfunction. Fluopsin C caused ROS accumulation and oncosis in the MCF-7 and MD-MBA-231 cell lines [9]. ROS are closely associated with mitochondria. Excessive ROS production occurs downstream of microvillar degradation and homotypic adhesion but upstream of actin reorganization, plasma membrane damage, and mitochondrial membrane permeabilization [10]. Porimin is a transmembrane protein with 118 amino acids; it belongs to the mucin family and is a specific receptor protein that mediates oncosis [8]. Once the Porimin protein and its ligand combine, they quickly break the integrity of the cell membrane, mediate the formation of pores in the cell membrane, increase cell membrane permeability, and cause cell swelling and death. Therefore, Porimin acts as an important marker of oncosis.

*Salvia miltiorrhiza* is used in traditional Chinese medicine. It is commonly used in clinics; has pharmacological effects such as anti-oxidation, anti-inflammation, and anti-fibrosis; is widely used for cardiovascular and nerve protection [11]. In recent years, the anti-tumor effects of *Salvia miltiorrhiza* have been reported [12]. Dihydrotanshinone (DHT) is a bioactive compound of *Salvia miltiorrhiza* that has anti-cancer activity in malignant tumors [13].

In this study, we found that DHT was able to induce Porimin-dependent oncosis via ROS-mediated mitochondrial dysfunction in vivo and in vitro, which indicates that DHT is a potential drug for the treatment of NSCLC.

## 2. Results

### 2.1. DHT Triggered A549 Cell Death in a Non-Apoptosis Type

**Figure 1:**
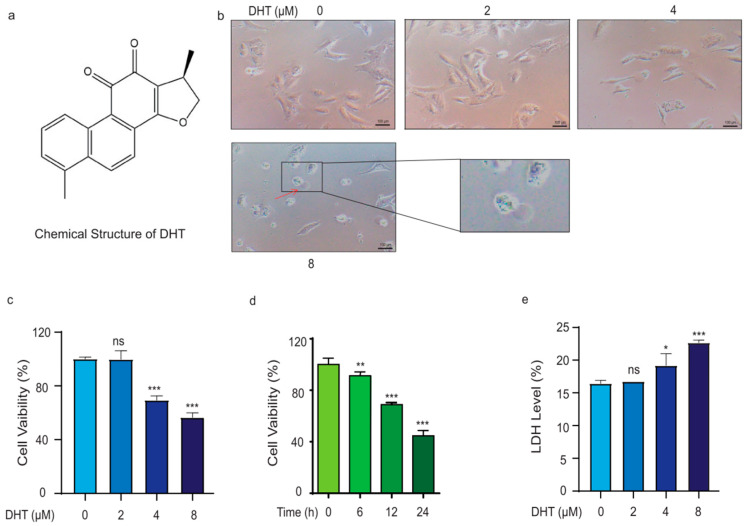
DHT triggered A549 cell death in a non-apoptosis type. (**a**) Structure of DHT. (**b**) The morphological changes in DHT on cells. The red arrow in the figure shows the cell bubbling (original magnification; scale bar = 100 µm). (**c**–**e**) Effect of DHT on cell viability and LDH activity in A549 cells. (*n* ≥ 3; ns means not statistically significant, * *p* < 0.05*,* ** *p* < 0.01*,* *** *p* < 0.001 vs. control group).

The structure of DHT is shown in Figure 1a. Compared to the control group, with the increase in drug concentration, the cell viability was decreased, the cell morphology changed significantly, and the cell membrane showed an obvious bubbling phenomenon (Figure 1b). The red arrow in the figure shows the cell bubbling. The cytotoxicity of DHT (Figure 1c,d) on A549 cells was determined by MTS assays. In addition, we also examined the cytotoxicity of DHT on H1299 and HLF cells (Figure S1). We found that DHT caused A549 cell death in a dose- and time-dependent manner. And DHT also caused H1299 and HLF cells death. The LDH release level of DHT (2, 4, and 8 μM) was analyzed (Figure 1e). Compared to the control group, DHT treatment significantly increased the level of LDH released by A549 cells. The results also showed that the number of cell deaths increased with the increase in drug concentration. These data suggested that DHT-induced cell death in A549 cells and the cell death type was different from apoptosis.

### 2.2. DHT-Induced, Porimin-Dependent Oncosis in A549 Cells

**Figure 2:**
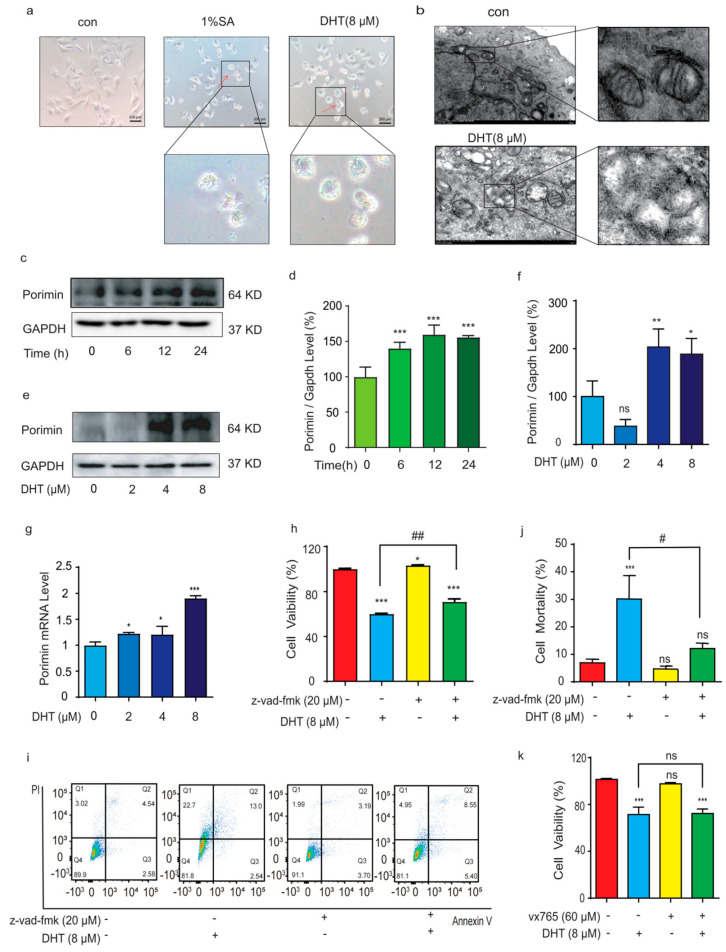
DHT-induced, Porimin-dependent oncosis in A549 cells. (**a**) The morphological changes in A549 cells after treatment with 1% SA or DHT (8 µM) for 24 h (original magnification; scale bar = 200 µm). Red arrows indicate oncosis cells (**b**) The features of oncosis in A549 cells were detected by TEM (scale bar = 2.0 µm). (**c**,**d**) The expression of Porimin at different times (6, 12, and 24 h) in A549 cells was detected by Western blotting. (**e**,**f**) The expression of Porimin in A549 cells induced by DHT (2, 4, and 8 µM) for 24 h was detected by Western blotting. (**g**) The expression of Porimin mRNA in A549 cells. (**h**) MTS assay was used to analyze the cell viability of the treatment with DHT (8 µM) for 24 h in the presence of z-vad-fmk (20 µM) in A549 cells. (**i**,**j**) Annexin V and PI double staining assay was used to analyze the effect of the treatment with DHT (8 µM) for 24 h in the presence of z-vad-fmk (20 µM) in A549 cells. The bar statistics of the PI positive cells rate. (**k**) MTS assay was used to analyze the effect of the treatment with DHT (8 µM) for 24 h in the presence of vx765 (60 µM) in A549 cells. (*n* ≥ 3; ns means not statistically significant, * *p* < 0.05*,* ** *p* < 0.01*,* *** *p* < 0.001 vs. control group. *^#^ p* < 0.05*,* ^##^ *p* < 0.01 vs. DHT (8 µM) group).

Recently, it has been documented that sodium azide (SA) can induce cell oncosis [14]. The red arrows are oncosis cells, and it can be observed that the cell swelling and bubbling in the DHT treatment were similar with 1% SA-induced oncosis (Figure 2a). It can be preliminarily found that, during DHT-induced oncosis in A549 cells, and compared with the control group, mitochondria appear to undergo densification in the early stage and swelling in the later stage, receive damage to the mitochondrial crista, and suffer a loss of function due to damage to the mitochondrial structure (Figure 2b).

Porimin is a specific receptor protein that mediates oncosis [8]. As shown in Figure 2c–f, compared with the control group, after A549 cells were treated with DHT for different times or concentrations, the expression of Porimin increased significantly. The expression of Porimin mRNA increased with the increase in DHT concentrations (Figure 2g). Compared to the control group, the z2212vad-fmk group was able to inhibit DHT-induced A549 cells (Figure 2h). As shown in Figure 2i,j, Annexin V and PI double staining results revealed that the cell death induced by DHT was increased. Inhibitor vx765 treatment did not reverse the cell death induced by DHT in A549 cells (Figure 2k). These data indicated that DHT-induced cell death was a result of Porimin-dependent oncosis.

### 2.3. ROS Regulated DHT-Induced, Porimin-Dependent Oncosis in A549 Cells

**Figure 3:**
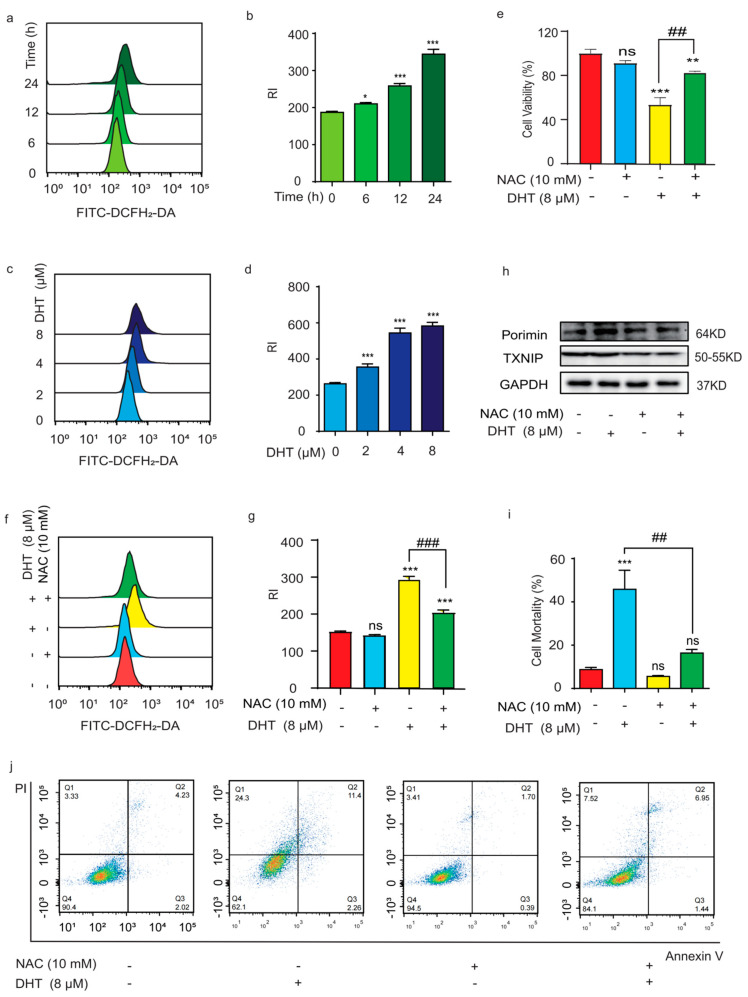
ROS regulated DHT-induced, Porimin-dependent oncosis in A549 cells. (**a**,**b**) After treating A549 cells with DHT (8 μM) for different amounts of time (6, 12, and 24 h), we added ROS probe DCFH_2_-DA (1 μM), incubated in darkness for 30 min, and then measured the ROS level of cells by flow cytometry. (**c**,**d**) After treating A549 cells with different concentrations of DHT (2, 4, and 8 μM) for 24 h, the ROS probe DCFH_2_-DA (1 μM) was incubated in dark for 30 min, and then we measured the ROS level of cells by flow cytometry. (**e**) The cell viability of A549 cells was tested by treatment with DHT (8 µM) and in the presence of NAC (10 mM). (**f**,**g**) ROS level was tested by treatment with DHT (8 µM) and in the presence of NAC (10 mM). (**h**) The expression of Porimin protein and TXNIP protein in A549 cells. (**i**,**j**) Annexin V and PI double staining assay was used to analyze the effect of the treatment with DHT (8 µM) for 24 h in the presence of NAC (10 mM) in A549 cells. (*n* ≥ 3; ns means not statistically significant, * *p* < 0.05, ** *p* < 0.01, *** *p* < 0.001 vs. control group. ^##^ *p* < 0.01, ^###^ *p* < 0.001 vs. DHT (8 µM) group).

ROS play a key role in oncosis, which often presents in mitochondrial dysfunction. High levels of ROS can lead to DNA damage, which induces cell death [15]. In this study, a DCFH_2_-DA probe was used to examine the ROS level in DHT-treated A549 cells. Compared to the control group, the ROS level of cells was significantly increased by the DHT treatment in a time- and dose-dependent manner (Figure 3a–d). It has been reported that ROS and its derivatives can cause oxidative damage to membrane proteins and inhibit the electron transport of the respiratory chain, thereby reducing ATP production and eventually leading to cell oncosis [16]. Compared to the control group, NAC can significantly ameliorate cell death (Figure 3e) and decrease cellular ROS levels induced by DHT (Figure 3f,g). In addition, NAC suppressed DHT-induced Porimin and TXNIP expression in A549 cells (Figure 3h). TXNIP is an endogenous inhibitor of trx, and trx is an antioxidant protein, so the levels of TXNIP and ROS are closely related [17]. Trx could reduce disulfides to thiol groups to control cellular reactive oxygen species [18]. TXNIP can bind to oxidized Trx2 in mitochondria, causing mitochondrial dysfunction [19]. The Annexin V and PI double staining results showed that NAC could reduce the PI-positive rate of A549 cells induced by DHT (8 µM). Collectively, these data indicated that ROS play an important role in DHT-induced, Porimin-dependent oncosis in A549 cells.

### 2.4. Mitochondria Depletion and Ca^2+^ Influx Regulated DHT-Induced, Porimin-Dependent Oncosis in A549 Cells

**Figure 4:**
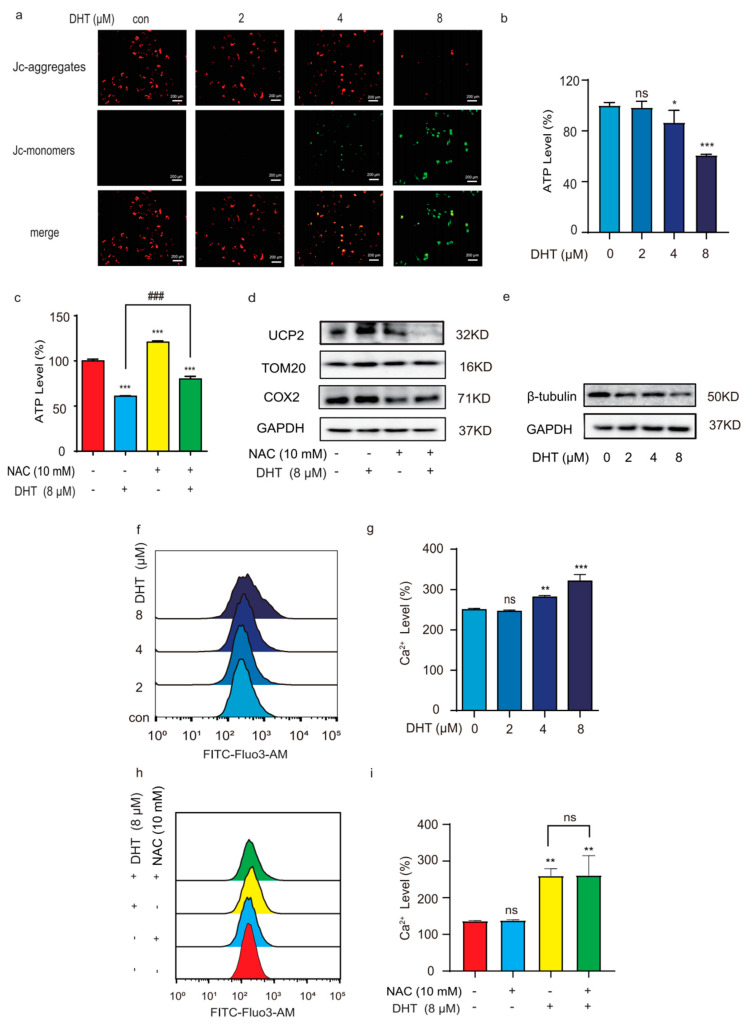
Mitochondria depletion and Ca^2+^ influx regulated DHT-induced, Porimin-dependent oncosis in A549 cells. (**a**) A JC-1 kit was used to examine the MMP levels in A549 cells (original magnification; scale bar = 200 µm). Normal mitochondrial membrane potential was shown in red with JC-1 aggregates and depolarized membrane potential was shown in green in JC-1 monomers. (**b**,**c**) ATP levels were tested using a multifunctional microplate reader in A549 cells. (**d**,**e**) A WB assay was used to detect protein expression of β-tubulin, UCP2, COX2, TOM20, and GAPDH in A549 cells. (**f**,**g**) A549 cells were treated with different concentrations of DHT (2, 4, and 8 μM) for 24 h, and then we tested the Ca^2+^ levels by flow cytometry. (**h**,**i**) After pretreatment with NAC (10 mM) for 1 h, A549 cells were treated with DHT (8 μM) for 24 h and incubated in darkness for 1 h with the Fluo-3AM. Ca^2+^ levels were tested by flow cytometry in A549 cells. (*n* ≥ 3; ns means not statistically significant, * *p* < 0.05, ** *p* < 0.01, *** *p* < 0.001 vs. control group. ^###^ *p* < 0.001 vs. DHT (8 µM) group).

Mitochondria were involved in many kinds of signal transduction, which provided energy for cells and produced ROS. The antioxidant system in mitochondria and reactive oxygen species antagonize each other to maintain the normal growth of cells. As shown in Figure 4a, the JC-1 staining results showed that DHT induced mitochondrial dysfunction in A549 cells. The aerobic metabolism and glycolysis of mitochondria are also related to tumor development. As shown in Figure 4b, compared to the control group, ATP levels were significantly decreased by treatment with DHT (8 µM). However, NAC decreased the ATP level induced by DHT in A549 cells (Figure 4c). The results showed that the increase in ROS induced by DHT in A549 cells consumed ATP, resulting in the destruction of mitochondrial function. UCP2 is a mitochondrial anion carrier protein that regulates ATP and ROS production [20]. In addition, ROS can also activate UCP2 [21]. There is evidence that a modest increase in UCP2 expression levels leads to a sharp decrease in mitochondrial membrane potential and a decrease in mitochondrial NADH and intracellular ATP, inducing oncosis [22]. Our results showed that DHT can increase the expression of UCP2 in A549 cells. After the intervention of NAC, the level of ROS was inhibited, and the level of UCP2 was decreased (Figure 4d). There is evidence that increased ROS levels can promote the aggregation of mitochondrial membrane protein TOM20 and the expression of COX2 [23,24,25]. Our results showed that DHT can increase the expression of TOM20 and COX2 in A549 cells (Figure 4d).

The influx of calcium ions may destroy the cytoskeletal proteins of the cell membrane, and, while it increases the permeability of the cell membrane, it did not regulate mitochondria dysfunction in DHT-induced oncosis (Figure 4e). Ca^2+^ is involved in multiple-signaling regulatory pathways within the cells. Ca^2+^ can promote the proliferation and metastasis of tumor cells [26]. The increase in intracellular Ca^2+^ can aggravate the consumption of ATP and promote cell oncosis. As shown in Figure 4f,g, we found that DHT can promote the increase in the Ca^2+^ level. However, in the presence of NAC (10 mM), there were no significant changes compared to the NAC pretreatment group (Figure 4h,i). These results indicated that mitochondrial depletion and Ca^2+^ influx play an important role in DHT-induced oncosis.

### 2.5. DHT-Induced Oncosis in LLC Xenograft Mice Model

**Figure 5:**
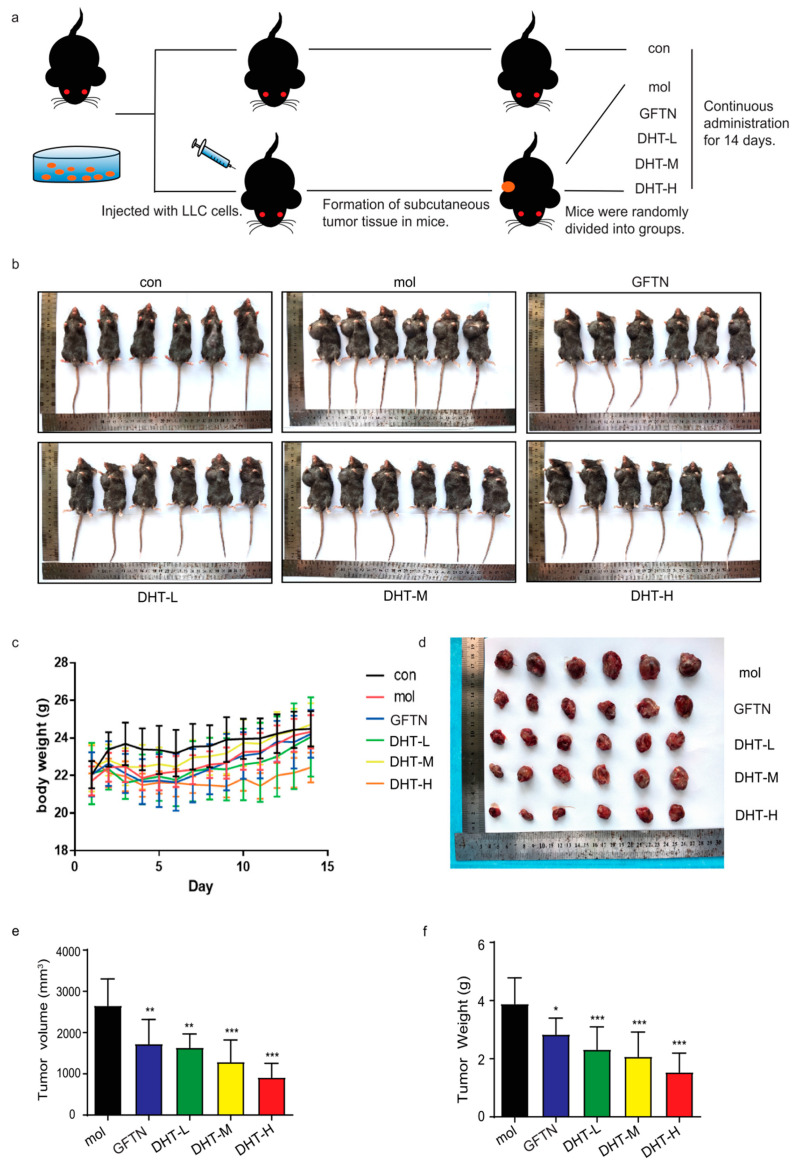
DHT-induced oncosis in LLC xenograft mice model. (**a**) The schematic diagram of the animal experiment. (**b**) Photograph of tumor in mice. (**c**) Statistical chart of body weight of mice. (**d**–**f**) Effect of DHT on tumor weight and volume. Compared to model group, ns means not statistically significant: * *p* < 0.05; ** *p* < 0.01; *** *p* < 0.001. *n* ≥ 6.

**Figure 6:**
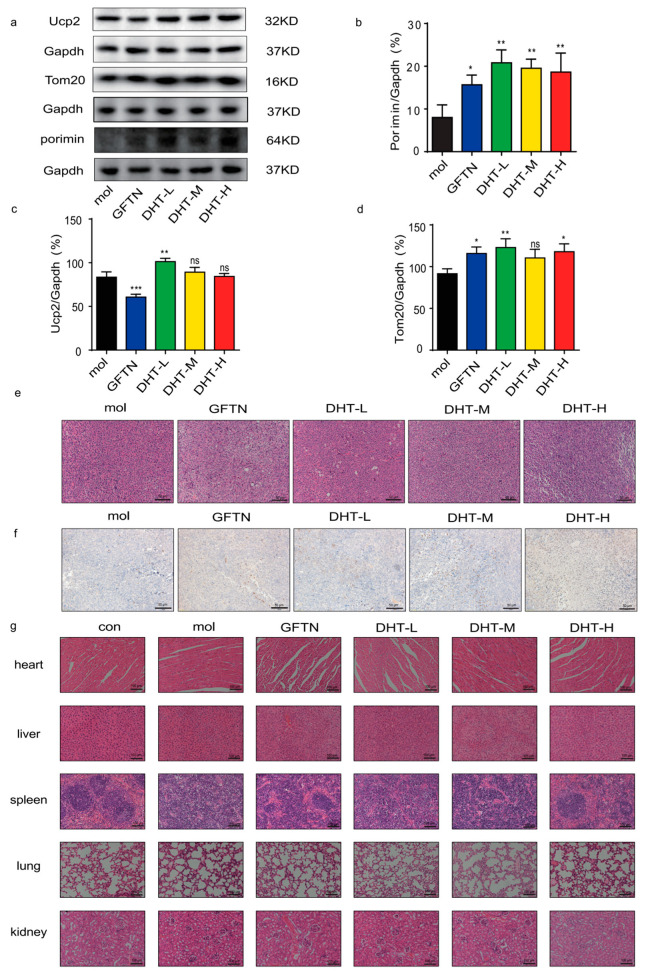
DHT-induced oncosis in lung cancer cells in mouse models. (**a**–**d**) Expression of Porimin, UCP2, and TOM20 proteins in tumor tissues of mice. (**e**) HE staining of tumor tissues. (HE, original magnification; scale bar = 50 µm). (**f**) Immunohistochemical staining of tumor tissues. (IHC, original magnification; scale bar = 50 µm). (**g**) HE staining of heart, liver, spleen, lung, and kidney tissues. (Scale bar = 100 µm). Compared to the model group, ns means not statistically significant: ns means not statistically significant, * *p* < 0.05; ** *p* < 0.01; *** *p* < 0.001. *n* ≥ 6.

In order to further examine the anti-cancer effect of DHT in vivo, we used LLC cells to construct a mouse xenograft model for evaluation (Figure 5a). As shown in Figure 5b, the tumor volume of each administration group was smaller than that of the model group. And the body weight showed no significant difference between the control group and model group (Figure 5c). As shown in Figure 5d–f, the tumor tissues of mice were taken, and their volume was measured. We found that the high dose of DHT significantly inhibited tumor growth in mice compared to the model group. Furthermore, compared to the model group, DHT administration increased the expression levels of Porimin, UCP2, and TOM20 proteins (Figure 6a–d). The HE staining of tumor tissues showed that DHT induced cell death (Figure 6e). The results of IHC showed that the expression of Porimin in the DHT group was more significant than that in the model group (Figure 6f). Compared with the control group, the heart, liver, lung, and kidney in DHT-treated groups had no significant damage. The spleen of mice in the model group had obvious pathological changes, while the gefitinib group and DHT high-dose group can effectively improve the pathological damage of the spleen of mice (Figure 6g). These results described that DHT-induced, Porimin-dependent oncosis in LLC xenograft mice model.

## 3. Discussion

Oncosis is a form of unprogrammed cell death characterized by cell swelling and karyolysis [7]. The morphological manifestations of oncosis mainly include cell enlargement, swelling, cell vesicles, a lack of organelles in the vesicles, the destruction of cell membrane integrity, the swelling of the endoplasmic reticulum, the swelling of mitochondria, a swollen nuclear membrane, dispersion, and the agglutination of chromatin [8,27,28,29,30]. In the later stage, the contents of the cells overflow, causing an inflammatory reaction [31,32]. The Porimin protein belongs to a family of membrane proteins comprising 118 amino acids and is specifically expressed on the surface of oncotic cells [33].

Lung cancer is one of the cancers with high morbidity and mortality, and the causes related to lung cancer incidence include smoking, occupational exposure (e.g., coal smoke), chronic obstructive pneumonia, family history, etc. [34]. Traditional chemotherapeutic drugs often induce the apoptosis of tumor cells, but the drug resistance of chemotherapeutic drugs has limited their clinical application in recent years [32,35]. Therefore, it was necessary to find effective therapeutic agents. Oncosis in NSCLC may provide a new idea for the treatment of NSCLC, especially for apoptosis-resistant NSCLC. For example, the cyclometalated Ir (III) complex activates the oncosis-specific proteins Poriminn and calpain in the cisplatin-resistant cell line A549R, inducing oncosis [36]. Aspirin induces oncosis in A549 cells by decreasing Bcl-XL levels and subsequent ATP depletion [37]. Interleukin-33 enhances the programmed oncosis of ST2L-positive low-metastatic cells in the tumor microenvironment of lung cancer [38].

DHT is one of the active components of *Salvia miltiorrhiza*, and it is cytotoxic to a variety of malignant tumors. DHT inhibits ovarian tumor growth by activating oxidative stress via Keap1-mediated Nrf2 ubiquitination and degradation [39]. However, the potential therapeutic mechanism of DHT for NSCLC is unclear [40,41,42]. In this study, we found that DHT could induce Porimin-dependent oncosis in A549 cells. And we found that DHT caused approximately 50% cell death in A549 cells. The literature reports that lactate dehydrogenase is one of the markers of cell oncosis [43,44,45]. Our results indicated that DHT increased the level of LDH in A549 cells. Western blotting experiments showed that, after the administration of DHT, the expression of Porimin significantly increased, which can rapidly bind to its ligand and destroy the structure of the cell membrane.

ROS can promote tumor growth, proliferation, migration, and invasion. On the other hand, excessive ROS can promote tumor cell death. ROS can activate the MAPK signaling pathway and promote cell apoptosis [46]. In cells, mitochondria were the main site of ROS production and the site of ROS action [47]. Our study found that DHT can increase the level of ROS released by cells. NAC could reverse the oncosis of A549 cells induced by DHT. NF-κB protects lung epithelia from hyperoxia-induced oncosis [48]. And the NF-κB pathway is upstream of ROS. Therefore, it is necessary to further study the relationship between the NF-κB pathway and oncosis. TXNIP is closely related to ROS, and it can indirectly reflect cell inflammation. We found that DHT increased the expression of TXNIP in A549 cells. TOM20 and UCP2 are mitochondria-associated proteins. A modest increase in UCP2 expression levels results in a rapid and dramatic decrease in mitochondrial membrane potential [22]. We found that DHT increased the expression of UCP2 in A549 cells. However, further studies need to explore the relationship between the UCP2 protein and oncosis.

Ca^2+^ was involved in oncosis-like cell death and participated in various intracellular signaling pathways to regulate cell growth and metabolism [49,50]. Ca^2+^ played a key role in mediating cPLA2-induced renal cell oncosis [51]. The mechanism by which Ca^2+^ is involved in oncosis is not well understood. Ca^2+^ is stored in the endoplasmic reticulum and can be taken up by mitochondria, which become overloaded, affecting mitochondrial oxidative phosphorylation, decreasing ATP production, and causing oncosis. Our study confirmed that DHT can induce an increase in calcium ion levels in A549 cells. In another, Ca^2+^ contributed to the remodeling of the actin cytoskeleton [52]. We found that DHT can induce the decrease in A549 cytoskeleton protein β-tubulin expression. Therefore, we speculate that Ca^2+^-induced cell death may be closely related to cytoskeletal proteins. However, the relationship between calcium and oncosis, such as in the use of calcium antagonists, needs to be further studied.

In this experiment, we selected C57BL/6J mice and constructed LLC tumor-bearing mice. DHT (40 mg/kg) significantly inhibited tumor growth in LLC-transplanted mice. The anti-cancer effect of DHT was better than that of the gefitinib group. Gefitinib is a commonly used clinical drug for NSCLC. It causes diarrhea, rash, vomiting, hepatotoxicity, and other adverse reactions. In our study, we found that DHT had anti-cancer activity and improved spleen damage. Therefore, DHT may be a potential anti-cancer drug. With the increase in the dose of DHT, the anti-tumor effect was stronger. The clinical application of gefitinib is limited due to its drug resistance. While DHT inhibits NSCLC tumor growth by inducing oncosis, the mechanism is different from gefitinib. This may provide a new strategy for attenuating gefitinib resistance in NSCLC. Consistently with in vitro results, DHT increased Porimin expression in LLC xenograft mouse tumor cells. This study will provide a type of cell death that is distinct from that found in in NSCLC following DHT-induced apoptosis, which may be helpful for the clinical chemotherapy treatment of drug-induced, apoptosis-resistant NSCLC cells.

## 4. Materials and Methods

### 4.1. Materials

DHT (purity ≥ 98%) was purchased from Chengdu Pufei De Biotech Co., Ltd. (Chengdu, China). MTS, N-Acetyl-L-cysteine (NAC), Ca^2+^ probe (Fluo-3/AM), and ROS probe (DCFH_2_-DA) were purchased from Sigma-Aldrich (St. Louis, MO, USA). z-vad-fmk and vx765 were purchased from Selleck Chemicals (Houston, TX, USA). COX-2, TOM20, and GAPDH were purchased from Cell Signaling Technology (CST, Danvers, MA, USA). Porimin was purchased from Bioss (Beijing, China). TXNIP was purchased from Proteintech (Chicago, IL, USA). UCP2 was purchased from Affinit (Cincinnati, OH, USA). β-tubulin was purchased from Abmart (Shanghai, China). LDH assay kit and 10% sodium azide were purchased from Beyotime Biotechnology (Shanghai, China). Mitochondrial membrane potential assay kit (JC-1) was acquired from Thermo Fisher Scientific (Waltham, MA, USA). BCA protein assay kit, PVDF membranes, and qRT-PCR kit were bought from Thermo Fisher (Waltham, MA, USA). Annexin V-EGFP apoptosis detection kit was bought from Shanghai Epizyme Biomedical Technology Co., Ltd. (Shanghai, China).

### 4.2. Cells and Cell Culture

A549 cells were purchased from the American Type Culture Collection (ATCC). Lewis lung carcinoma cells (LLC) were purchased from the Type Culture Collection of the Chinese Academy of Sciences. DMEM, RPMI 1640, trypsin, and FBS were purchased from Life Technologies/Gibco Laboratories (Grand Island, NY, USA). A549 cells were cultured in an RPMI 1640 medium supplemented with 10% FBS, penicillin (100 IU/mL), and streptomycin (100 μg/mL). LLC cells were cultured in DMEM medium with 10% fetal bovine serum (FBS), penicillin (100 IU/mL), and streptomycin (100 μg/mL). All cells were cultured in a humidified incubator under 5% CO_2_ at 37 °C.

### 4.3. Cell Viability Assay

DHT was dissolved in DMSO and diluted to 2 μM, 4 μM, and 8 μM with an RPMI 1640 medium containing 10% FBS. A549 cells were seeded in a 96-well cell culture plate with 5 × 10^3^ cells/well for overnight growth and then treated with DHT of different concentrations for 24 h. MTS reagent diluted 1/10 with an RPMI 1640 medium containing 10% FBS and cultured for 1 h. Subsequently, the absorbance value was measured at 490 nm with a microplate reader (SYNERGYH1, Bio Tek, Winooski, VT, USA).

### 4.4. Inhibitors Treatment

A549 cells were seeded in a 96-well cell culture plate with 5 × 10^3^ cells/ well for overnight growth and then replaced with a medium containing 1% FBS. After pre-application with apoptosis inhibitor z-vad-fmk (20 μM) for 1h, pyroptosis inhibitor vx765 (60 μM) for 1 h, or NAC (10 mM) for 1 h, the A549 cells were treated with DHT (8 μM) for another 24 h. MTS assays were used to detect cell viability.

### 4.5. LDH Detection

A549 cells were seeded in a 96-well cell culture plate with 5 × 10^3^ cells/well for overnight growth, treatment with or without prior addition of inhibitor, and then treatment with DHT of different concentrations for 24 h. The supernatant was collected according to the instructions of the manufacturer of the LDH detection kit, and the LDH level was evaluated by detecting the absorbance at 490 nm with a microplate reader (SYNERGYH1, Bio Tek, Winooski, VT, USA).

### 4.6. Intracellular ATP Detection

A549 cells were seeded in a 12-well cell culture plate with 1 × 10^5^ cells/well for overnight growth, treatment with or without prior addition of inhibitor, and then treatment with DHT of different concentrations for 24 h. Luminescence value (Lum) was detected by a microplate reader (SYNERGYH1, Bio Tek, Winooski, VT, USA) according to the instructions of the ATP detection kit.

### 4.7. Analysis of Mitochondrial Membrane Potential

A549 cells were seeded in a 96-well cell culture plate with 5 × 10^3^ cells/well for overnight growth and then treated with DHT of different concentrations for 24 h. Mitochondrial membrane potential (MMP) was measured according to the JC-1 kit instructions. The cells were washed with appropriate PBS, incubated with JC-1 solution (10 µM) for 1 h, observed, and photographed with a fluorescence microscope (Leica, Wetzlar, Germany).

### 4.8. Cell Death Detection

The cell death rate was detected according to the instructions of Annexin V-EGFP apoptosis detection kit. A549 cells were seeded in a 96-well cell culture plate with 6 × 10^5^ cells/well for overnight growth, treatment with or without prior addition of inhibitor, and then treatment with DHT of different concentrations for 24 h. The cells were collected, washed with cold PBS, and stained in the dark according to the instructions. Then, cell death was detected with a flow cytometer (Becton-Dickinson, Bedford, MA, USA).

### 4.9. Flow Cytometry

A549 cells were seeded in a 96-well cell culture plate with 6 × 10^5^ cells/well for overnight growth, treatment with or without prior addition of inhibitor, and then treatment with DHT of different concentrations for 24 h. The cells were stained with DCFH_2_-DA probe (5 μM) for 30 min or Fluo-3/AM (5 μM) for 1 h at 5% CO_2_ and 37 °C. The medium was purged and washed with cold PBS, and cells were collected. Then, Changes in cellular ROS and Ca2^+^ were detected with a flow cytometer (Becton-Dickinson, Bedford, MA, USA).

### 4.10. Western Blotting Analysis

A549 cells were seeded in a cell culture dish with 6 × 10^5^ cells/well for overnight growth, treatment with or without prior addition of inhibitor, and then treatment with DHT of different concentrations for 24 h. Cells were lysed using RIPA lysis buffer containing 1% cocktail and 1% PMSF. The proteins quantitative were tested by a BCA protein kit. The proteins were separated by SDS-PAGE and transferred to PVDF membrane. Then, 10% skimmed milk was used to seal the membrane for 2 h. The primary antibody (1:1000) was incubated overnight at 4 °C, and then the secondary antibody (1:5000) was incubated at room temperature by shaking the table for 2 h.

### 4.11. qPCR Analysis

A549 cells were seeded in a 30 mm cell culture dish with 6 × 10^5^ cells/well for overnight growth and then treated with DHT of different concentrations for 12 h. Total RNA was extracted using Trizol reagent. Complementary DNA (cDNA) was synthesized by the PrimeScript RT Reagent Kit. RNA amplification was performed according to the SYBR green qPCR master mix kit instructions. The gene sequences used were as follows:

Porimin-F: 5′-GCGGCATCTAATACAACACCAG-3′;
Porimin-R: 5′-TTGTGGGTTACGGTCATTGTGGATG-3′;
GAPDH-F: 5′-CATGACCACAGTCCATGCCATCAC-3′;
GAPDH-R: 5′-TGAGGTCCACCCACCCTGTTGCTGT-3′.

### 4.12. Transmission Electron Microscopy

A549 cells with 1 × 10^6^ cells per well were seeded in the cell culture dish and then treated with DHT of different concentrations for 24 h. The cells were washed with PBS and fixed with an electron microscope fixative. Place the cell sample on the copper mesh for staining and observe and take photos under the electron microscope (Hitachi).

### 4.13. Animal Experiment

Animal experiments were approved by the Ethics Committee on Laboratory Animal Management of Guangxi University of Chinese Medicine (approval document No. SYXK-2019-0001). All animals received humane care according to the local guidelines for the Care and Use of Laboratory Animals of the Guangxi University of Chinese Medicine. Healthy SPF grade C57BL/6JNifdc mice (male, 19–22 g, and 6–8 weeks old) were purchased from Beijing Vital River Laboratory Animal Technology Co., Ltd. (Beijing, China, animal license #: SCXK-2021–0006). All animals were fed under standard SPF-grade conditions at 25 °C with 50% relative humidity, conventional feeding, and free drinking water. LLC cells (5 × 10^5^ cells/mouse) were injected into the armpit of the right forelimb of mice except for the control group. After tumorigenesis, mice were randomly divided into a blank control group, a model group (normal saline), a GFTN (40 mg/kg) group, a first DHT (10 mg/kg) group, a second DHT (20 mg/kg) group, and a third DHT (40 mg/kg) group (*n* = 8). The DHT groups and the model group were administered by intraperitoneal injection, while the GFTN group was administered by intragastric administration. All drugs were dissolved in normal saline and administered once a day for 14 consecutive days. The tumor volume was measured every 2 days. The calculation formula of tumor volume is V = (A × B^2^)/2. (A is the longest diameter of the tumor, and B is the shortest diameter of the tumor).

### 4.14. Histopathology Analysis

The main organs of the mouse (heart, liver, spleen, lung, kidney, and tumor) were collected for HE and IHC staining and observed and photographed with an upright microscope.

### 4.15. Statistics

Statistical analysis was performed with GraphPad Prism 8.0 Software. Comparisons between variables were performed with a one-way ANOVA test. The results were considered to be significantly different when *p* < 0.05.

## 5. Conclusions

This study evaluated DHT-induced, Porimin-dependent oncosis in NSCLC via ROS-mediated mitochondrial dysfunction and Ca^2+^ influx. DHT suppressed the LLC xenograft mice tumor growth by inducing Porimin-dependent oncosis. This study suggests that DHT has the potential to treat NSCLC.

## Data Availability

The data that support the findings of this study are available from the corresponding author upon reasonable request.

## Supplementary Materials

The following supporting information can be downloaded at: https://www.mdpi.com/article/10.3390/ijms241511953/s1.
