# Supplementary material for: Dihydrotanshinone Triggers Porimin-Dependent Oncosis by ROS-Mediated Mitochondrial Dysfunction in Non-Small-Cell Lung Cancer
Source: Int J Mol Sci. 2023 Jul 26;24(15):11953. doi: 10.3390/ijms241511953 (PMC10419281; doi:10.3390/ijms241511953)
Supplement: Supplementary file 1 [file ijms-24-11953-s001.zip › ijms-2413000-supplementary.pdf]

Supplementary material:

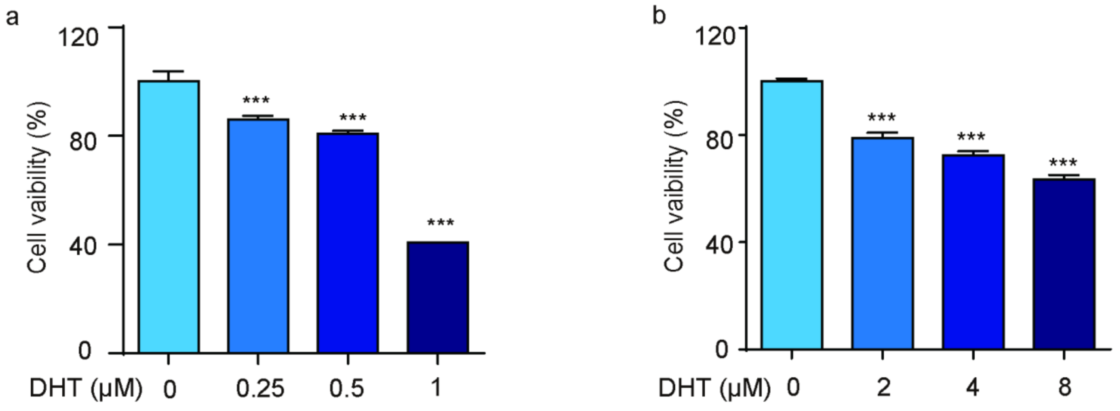


**Supplementary Figure S1. DHT inhibited the activity of H1299 cells and HLF cells. a** H1299 cells were treated with different concentrations of DHT (0.25, 0.5, 1 μM) for 24 hours, and then the cell activity was detected by microplate reader. **b** HLF cells were treated with different concentrations of DHT (2、4、8 μM) for 24 hours, and then the cell activity was detected by microplate reader. (n≧3, $^{***}$*p<0.001* vs DHT (0 μM) group. )
